# Excretion of Urotropin in Cerebrospinal Fluid; Its Therapeutic Value in Meningitis

**Published:** 1909-06

**Authors:** S. J. Crowe

**Affiliations:** Hunterian Laboratory of Experimental Medicine; *Johns Hopkins Hospital Bulletin*, April, 1909


### ABSTRACT.

### Excretion of Urotropin in Cerebrospinal Fluid; its Therapeutic Value in Meningitis.

By S. J. CROWE, M. D.
Hunterian Laboratory of Experimental Medicine; *Johns Hopkins Hospital Bulletin*, April, 1909.

In a prior publication the author reported a case with cerebrospinal fistula in which a fatal outcome was looked for, and in which at Dr. Cushing's suggestion, urotropin in large doses was given in the hope of its cerebrospinal excretion. The boy recovered; and this led to the writer's further studies. Thus in cases of chronic nephritis, brain tumor or hydrocephalus, when lumbar puncture was to be done, the patients were given a 10- or 15-grain dose of urotropin; the cerebrospinal fluid removed was tested by a modification of Hehner's test, and urotropin was invariably found present. A number of observations made showed that the drug may possibly be absorbed as readily by the rectum as when given by the mouth.

In some instances urotropin was not given till the cerebrospinal fluid was dripping steadily from the needle. By testing specimens every few minutes, the first appearance, maximum concentration and gradual disappearance of the drug could be followed. After a 15-grain dose, the maximum concentration generally appeared thirty minutes to one hour later.

To determine if the amounts which appeared in the cerebrospinal fluid were sufficient to exert decided bactericidal action, the fluid taken from the body was inoculated with organisms and the inhibitive effect noted. The tables and photographic plates incorporated in the paper show a striking antibacterial effect, namely a reduction in the number of colonies from about 30,000 to about 500 within 1 hour 50 min.

The effect of urotropin on meningitis in animals, produced by subdural inoculation with streptococci, was to defer or entirely prevent death.

Though there is no accurate method of determining the amount of urotropin in the fluids, the impression was gained, from the comparative color tests of cerebrospinal fluid and urine, that in the case of animals with meningitis a larger amount of urotropin appears in the cerebrospinal fluid than in the urine. If this be proved, it would suggest some selective action, possibly through the agency of the leukocytes, which have the power of taking up urotropin, as Vindevogel (*Ann. Soc. Roy. Scienc. Med.*, Bruxelles, 1902, Vol. II) proved.

When the first dose of urotropin was given subsequent to inoculation of the animals, the infection was merely checked during the continuance of active therapy; as soon as the drug was withdrawn, the organisms began to multiply with a return of clinical symptoms. This is much the same experience as with urotropin in genitourinary infections, and is akin also to the author's experiences with the drug in biliary infections. Therefore the most marked therapeutic value of the drug seems for prophylaxis or at least in early infections.

During the past year it has become a routine measure in the Johns Hopkins Hospital to promptly administer urotropin to all patients with lesions, which are not infrequently followed by meningeal infection; and the complete absence of such complication in quite an extensive series of cases seems to fairly well establish the prophylactic importance of the drug. This series included a number of compound fractures of the skull, gunshot wounds of the head and cerebrospinal fistulas, the patients receiving 30 to 60 grains urotropin daily. It is also used prior to ventricular or lumbar puncture, when local conditions make possible the inoculation of the meninges with organisms from an infected skin; just so urotropin should be given before a first catheterisation or one done when urethral infection is present. Possibly, too, the drug may be wisely used in cases of extracranial infection when extension to the meninges is feared, as in infected scalp wounds, otitis media, suppuration of the cranial sinuses. Its use may be desirable also in elaborate spinal or cerebral operations.

The author summarises: (1) Urotropin, given by mouth, invariably appears in the cerebrospinal fluid. This fact has been demonstrated by a large number of observations on man, and is also true for dogs and rabbits; (2) The largest amount of urotropin is present in the cerebrospinal fluid from 30 to 60 minutes after ingestion of the drug; (3) after therapeutic doses, a sufficient amount of urotropin appears in the cerebrospinal fluid to exercise a decided inhibitory effect on the growth of organisms inoculated into this fluid after removal from the body; (4) following a subdural inoculation of dogs and rabbits with streptococci, 60 to 80 grains of urotropin a day, given under conditions which insure absorption, will markedly defer, and in some cases prevent, the onset of a fatal meningitis; (5) the prompt administration of urotropin is advised in all clinical cases in which meningitis is a possible complication, or even when meningeal infection has actually occurred.

